# Identification of the changes in the platelet proteomic profile of elderly individuals

**DOI:** 10.3389/fcvm.2024.1384679

**Published:** 2024-05-14

**Authors:** Hui-Lian Chen, Qing-Yu Wang, Ruo-Mei Qi, Jian-Ping Cai

**Affiliations:** ^1^ The Key Laboratory of Geriatrics, Beijing Institute of Geriatrics, Institute of Geriatric Medicine, Chinese Academy of Medical Sciences, Beijing Hospital/National Center of Gerontology of National Health Commission, Beijing, China; ^2^ Graduate School of Peking Union Medical College and Chinese Academy of Medical Sciences, Beijing, China

**Keywords:** proteomics, platelets, ageing, inflammation, cardiovascular diseases

## Abstract

**Background:**

Platelet hyperreactivity is a risk factor for thrombosis in elderly patients with cardiovascular diseases. However, the mechanism of platelet hyperactivation has not been elucidated. This study aims to investigate alterations in the proteomes of platelets and their correlation with platelet hyperreactivity among elderly individuals.

**Methods:**

This study included 10 young (28.1 ± 1.9 years), 10 middle-aged (60.4 ± 2.2 years), and 10 old (74.2 ± 3.0 years) subjects. Washed platelets were used in the present study. Platelet samples were analysed by using data-independent acquisition (DIA) quantitative mass spectrometry (MS).

**Results:**

The results showed that the platelet proteomic profile exhibited high similarity between the young and middle-aged groups. However, there were significant differences in protein expression profiles between the old group and the young group. By exploring the dynamic changes in the platelet proteome with ageing, clusters of proteins that changed significantly with ageing were selected for further investigation. These clusters were related to the initial triggering of complement, phagosome and haemostasis based on enrichment analysis. We found that platelet degranulation was the major characteristic of the differentially expressed proteins between the old and young populations. Moreover, complement activation, the calcium signalling pathway and the nuclear factor-κB (NF-κB) signalling pathway were enriched in differentially expressed proteins.

**Conclusions:**

The present study showed that there are obvious differences in the protein profiles of the elderly compared with young and middle-aged populations. The results provide novel evidence showing changes in platelet hyperactivity and susceptibility to thrombosis in the elderly population.

## 1 Introduction

Platelets play a critical role in the progression of atherosclerosis and cardiovascular events. Altered platelet function is associated with a variety of chronic diseases, such as cardiovascular diseases, hypertension and diabetes. Growing evidence has demonstrated that the elderly population has increased platelet activity, which is manifested as thrombus susceptibility. Platelets are conventionally regarded as playing a vital role in haemostasis and thrombosis (1). This role has, over the years, transformed as our knowledge regarding platelets has expanded to include their role in inflammation, cancer progression, and metastasis. Recently, the fact that platelets are immune cells has been well established. Platelets are not only innate immune and inflammatory cells themselves, but they can also assist in adaptive immunity depending on the circumstances (2).

Elderly individuals have a higher risk of cardiovascular disease associated with platelet hyperactivity (3). Age-related vascular inflammation and platelet hyperactivity are linked to cardiovascular events, especially atherosclerosis, which is a chronic inflammatory disorder involving many immune cells, including platelets. Vascular endothelial cell injury, accompanied by endothelial cell activation, is the initial stage of atherosclerosis. Platelets are involved in the initiation and progression of atherosclerosis through the recruitment of inflammatory cells. In particular, activated platelets promote the adhesion of leucocytes to activated endothelial cells (4). Furthermore, platelets also play a dominant role in the formation of pathogenic thrombi in patients with atherosclerosis (5). At present, an increasing number of studies focus on the significance of platelet hyperactivity in elderly individuals. Multiple studies have reported that platelet counts decrease with age (6), while the platelet reactivity generally increases (7). Recently, a study found that elderly individuals had higher basal platelet activation than young individuals, which correlated with the increase in inflammation in elderly individuals (8). Additionally, cytokines such as β-thromboglobulin and platelet factor 4 promote thrombosis formation and are released by activated platelets in the plasma of elderly individuals (9). Le Blanc et al. reviewed the biochemical changes of platelets during the aging process and discussed the mechanisms by which these alterations may contribute to thrombotic diseases (10). Winkler et al. used two-dimensional DIGE to investigate the technical and total variation experienced in the analysis of platelet proteome in 20 healthy human volunteers aged 56–100 years (11). In general, platelet hyperactivity might be one of the key reasons for the increased risk of thrombosis in elderly individuals. However, the underlying mechanism of platelet hyperactivity in elderly individuals is less understood.

The use of proteomics by mass spectrometry has great potential in identifying and quantifying thousands of proteins from the smallest amount of material. Studies on platelet proteomics comprehensively describe the composition and copy numbers of human platelets, including platelet releasate (12–14). The development of platelet proteomics technology has greatly contributed to the study of platelets and their correlation with diseases, such as cognitive decline (15) and early-stage cancer (16). The recent emergence of data-independent acquisition (DIA) represents a major advance in protein quantification and is significant due to its capacity to analyse high-throughput quantitative proteomics data. However, platelet proteomic evidence regarding platelet hyperactivity is limited. This study aimed to evaluate age-related changes in the platelet proteome through DIA and investigate the association with platelet hyperactivity in elderly individuals.

## 2 Materials and methods

### 2.1 Subjects

A total of 30 participants were enrolled in the study, including 10 young (28.1 ± 1.9 years), 10 middle-aged (60.4 ± 2.2 years) and 10 old individuals (74.2 ± 3.0 years), with 5 males and 5 females in each group. The schematic of this study is shown in Supplementary Figure S1. The inclusion criteria were as follows: (1) age >18 years and signed informed consent form; (2) no mental disorders; and (3) no history of alcohol or drug abuse. The exclusion criteria included acute medical treatment or hospitalization within the first 3 months prior to blood collection; the chronic use of medications (e.g., aspirin and clopidogrel) and dietary supplements (e.g., fish oil and evening primrose oil) that may affect platelet function; the presence of severe diseases, including acute heart, liver, kidney disease, blood disease, or respiratory failure; and poorly controlled hypertension, diabetes or hyperlipidaemia. The characteristics of the subjects are shown in Table 1.

**Table 1:** Information for subjects.

Characteristics	Young (*n* = 10)	Middle (*n* = 10)	Old (*n* = 10)
Age, mean (SD), year	28.1 (1.9)	60.4 (2.2)	74.2 (3.0)
BMI, mean (SD)	23.8 (3.1)	24.1 (3.4)	23.6 (3.8)
Platelet count, mean (SD), 10^9 ^/L	247.3 (56.9)	251.8 (64.4)	221.5 (52.8)
SBP, mean (SD), mmHg	125.0 (14.6)	119.3 (22.2)	124.2 (15.7)
DBP, mean (SD), mmHg	84.3 (9.9)	81.5 (10.9)	78.2 (10.9)
Glucose, mean (SD), mmol/L	4.7 (0.5)	5.3 (0.4)	6.0 (1.5)
TG, mean (SD), mmol/L	1.0 (0.9)	1.5 (0.6)	1.7 (1.1)
TC, mean (SD), mmol/L	4.7 (0.7)	5.4 (0.5)	4.6 (0.9)
HDL, mean (SD), mmol/L	1.5 (0.2)	1.5 (0.4)	1.2 (0.3)
LDL, mean (SD), mmol/L	3.0 (0.5)	3.5 (0.4)	3.0 (0.6)

BMI, body mass index; SBP, systolic blood pressure; DBP, diastolic blood pressure; TG, triglyceride; TC, total cholesterol; HDL, high-density lipoprotein; LDL, low density lipoprotein.

### 2.2 Platelet preparation

The blood was collected from subjects through peripheral venipuncture into vacuum tubes containing 3.2% sodium citrate. The platelet-rich plasma (PRP) was obtained by centrifugation at 200 × g for 10 min within one hour after blood collection, and the upper 2/3 of the PRP was placed in a new tube. The PRP was then centrifuged at 300 × g for 10 min in the presence of acid-citrate-dextrose (ACD) and 10 μg prostaglandin *I*_2_ sodium salt (Aladdin, Shanghai, China), washed twice with modified Tyrode's buffer (138 mM NaCl, 3.3 mM NaH_2_PO_4_▪2H_2_O, 1 mM MgCl_2_, 2.9 mM KCl, 5.5 mM glucose, and 20 mM HEPES) to obtain purified platelet samples and stored at −80°C.

Protein extraction and peptide preparation were conducted by Novogene Co., Ltd. Briefly, platelet samples were completely lysed with DB lysis buffer [8 M urea, 100 mM triethylammonium bicarbonate (TEBA), pH 8.5], followed by 5 min of ultrasonication on ice. The lysate was centrifuged at 12,000 × g for 15 min at 4°C, and the supernatant was added to 1 M DL-dithiothreitol to react for 1 h at 56°C; the sample was subsequently alkylated with sufficient iodoacetamide for 1 h at room temperature in the dark followed by incubation in an ice bath for 2 min. Each protein sample was taken and the volume was made up to 100 μl with DB lysis buffer, trypsin and 100 mM TEAB buffer were added, sample was mixed and digested at 37°C for 4 h. Then, trypsin and CaCl_2_ were added and the samples were digested overnight. Formic acid was mixed with the digested sample, the sample was adjusted to a pH under 3, and the sample was centrifuged at 12,000 × g for 5 min at room temperature. The supernatant was slowly loaded onto the C18 desalting column, washed with washing buffer (0.1% formic acid, 3% acetonitrile) 3 times, and then elution buffer (0.1% formic acid, 70% acetonitrile) was added. The eluents of each sample were collected and lyophilized.

### 2.3 Western blot assay

Washed platelets were obtained using the same method as in 2.2 section. Whole cell lysates were separated by sodium dodecyl sulfate-polyacrylamide gel electrophoresis (PAGE) and transferred to polyvinylidene membranes. The membranes were incubated overnight at 4°C with specific primary antibodies (diluted to 1:1000) and then incubated with anti-mouse or anti-rabbit antibodies (diluted to 1:5000). The bands were exposed using electrochemiluminescent reagent and the EvolutionCapt system (Vilber Lourmat), and quantified using ImagePro Plus software. Monoclonal anti-macrophage migration inhibitory factor (MIF) antibody, polyclonal anti-mannan binding lectin serine peptidase (MASP) 1 antibody, monoclonal anti-superoxide dismutase (SOD) 1, Monoclonal anti-calumenin antibody were purchased from Zen-bioscience (Chengdu, China). Monoclonal anti-factor H antibody were purchased from Huaan Biotechnology (Hangzhou, China). Monoclonal anti-autophagy-related 7 (ATG7) and monoclonal anti-actin antibody were purchased from ABclonal (Wuhan, China).

### 2.4 SOD detection in plasma

The levels of SOD and other biochemical indexes in plasma were detected by KingMed Diagnostics (Guangzhou, China).

### 2.5 DIA MS analysis and data analysis

DIA MS analysis was conducted by Novogene Co., Ltd. (Beijing, China). LC-MS/MS spectra were searched using an EASY-nLCTM 1,200 UHPLC system coupled with an Orbitrap Q ExactiveTM HF-X mass spectrometer (Thermo Fisher, Germany) operating in DIA mode. Data analysis and visualization of DIA data were conducted by Novogene Co., Ltd. using the Proteome Discoverer 2.2 (PD 2.2, Thermo Fisher Scientific) platform, Biognosys Spectronaut v. 9.0, and R statistical framework. MS2-based label-free quantification was carried out by analysing DIA raw data using Biognosys Spectronaut v.9.

The Spectronaut-Pulsar further filtered the retrieval results, and peptide spectrum matches (PSMs) with credibility of more than 99% were identified PSMs. The identified protein contains at least 1 unique peptide. The identified PSMs and proteins were retained and analyzed with a false discovery rate (FDR) of no more than 1.0%. The proteins whose quantitation significantly differed between the experimental and control groups were evaluated by *t*-test [*p* < 0.05 and |fold change (FC)| > 1.5] were defined as differentially expressed proteins (17, 18). R (R-3.4.3) with the heatmap gplots package was used for C-means cluster analysis, heatmap drawing, and KEGG enrichment analysis. Metascape (http://metascape.org) was used for pathway and functional analyses. Cytoscape 3.9.1 and the STRING (v10; https://string-db.org/) plug-in were used for visual analysis of the protein-protein interaction (PPI) network. Gene set enrichment analysis (GSEA) 3.0 was used to conduct GSEA. Enrichment dot bubbles and bars with colour gradients were plotted by https://www.bioinformatics.com.cn (last accessed on 20 Feb 2023), an online platform for data analysis and visualization. The flow chart was drawn by Figdraw.

## 3 Results

### 3.1 Platelet proteomic analysis

**Figure 1:**
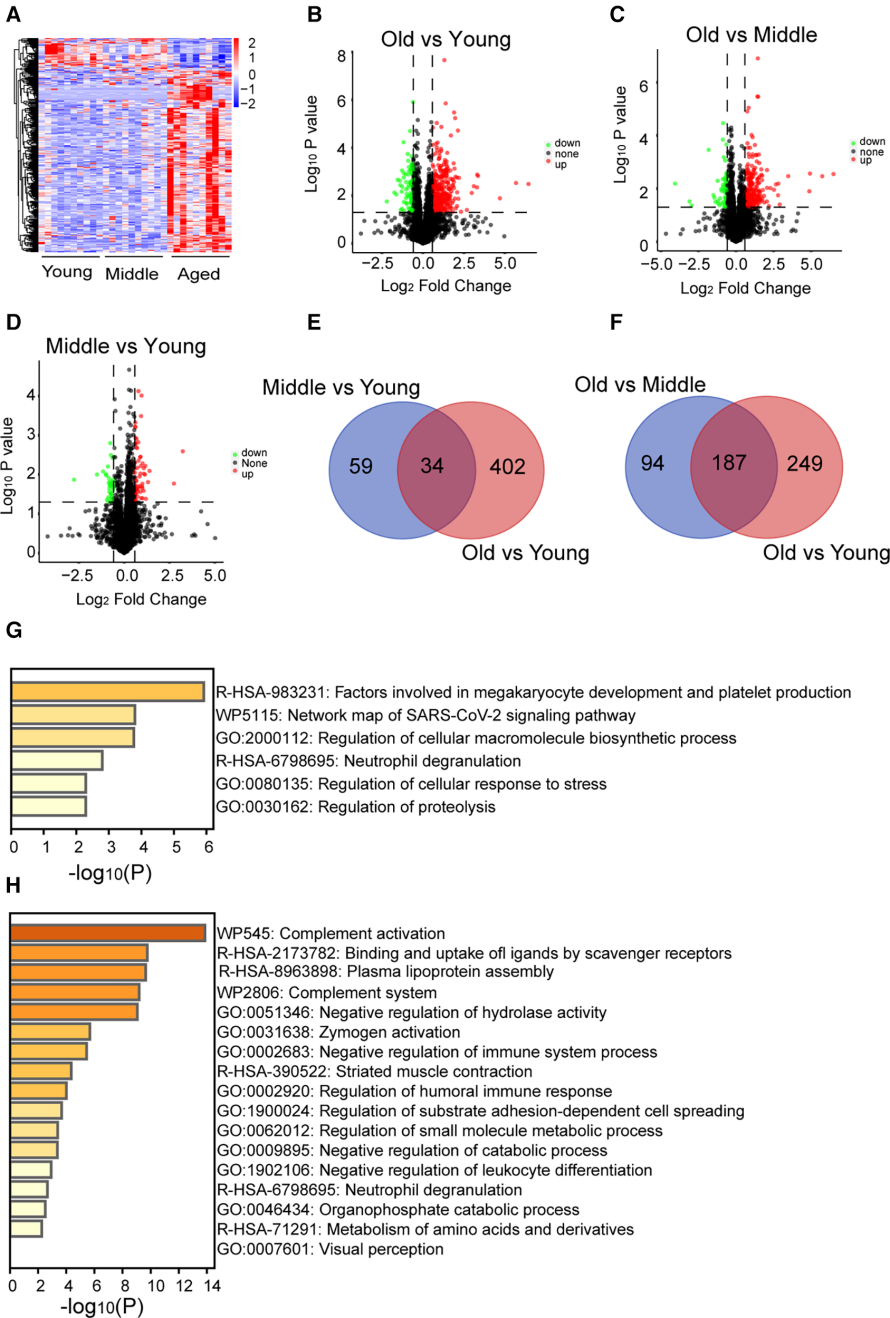
General proteome information. (**A**) A total of 580 differentially expressed proteins were identified in the platelets of the middle-aged and old groups compared with the young group (*p* < 0.05, increased proteins: red; decreased proteins: blue). (**B**–**D**) The increased (red) or decreased (green) level of proteins in the old vs. young, old vs. middle, or middle vs. young groups (*p* < 0.05). (**E**,**F**) Overlapping proteins in the old vs. middle and old vs. young, or middle vs. young and old vs. young groups. (**G**,**H**) Pathway enrichment analysis of overlapping proteins in the old vs. middle and old vs. young, or middle vs. young and old vs. young groups determined by Metascape online analysis.

In this study, we conducted proteomic analysis of platelet samples from 10 young, 10 middle-aged, and 10 elderly adults. A total of 44,756 peptides were detected, and 5,142 proteins were identified. Platelet proteomic analysis across different age groups demonstrated significant alterations in the protein profile of platelets from older individuals (Figure 1A; Supplementary Figure S2). Specifically, 332 differentially expressed proteins were upregulated (*p* < 0.05) and 104 were downregulated (*p* < 0.05) in the old group compared with the young group (Figure 1B), 227 differentially expressed proteins were upregulated (*p* < 0.05) and 54 were downregulated (*p* < 0.05) in the old vs. middle groups (Figure 1C), and 60 differentially expressed proteins were upregulated (*p* < 0.05) and 33 were downregulated (*p* < 0.05) in the middle vs. young groups (Figure 1D). Thirty-four differentially expressed proteins overlapped in both the middle vs. young and the old vs. young groups (Figure 1E). Enrichment analysis revealed that the overlapping 34 differentially expressed proteins, including beta-globin gene (HBB), human beta-defensins (HBD), TUBAL3, TUBB2B, and inter-alpha-trypsin Inhibitor Heavy chain 3 (ITIH3), were associated with “megakaryocyte development and platelet production” (Figure 1G). One hundred eighty-seven differentially expressed proteins overlapped in both the old vs. young and the old vs. middle groups (Figure 1F). The top enriched terms among the shared proteins included “complement activation”, among others (Figure 1H). These whole-proteome data reveal the differentially expressed proteins and the pathways involved in ageing. The list of differential proteins and overlapped proteins can be found in Supplementary Data Sheet 1.

### 3.2 Dynamic changes in the platelet proteome during the ageing process

**Figure 2:**
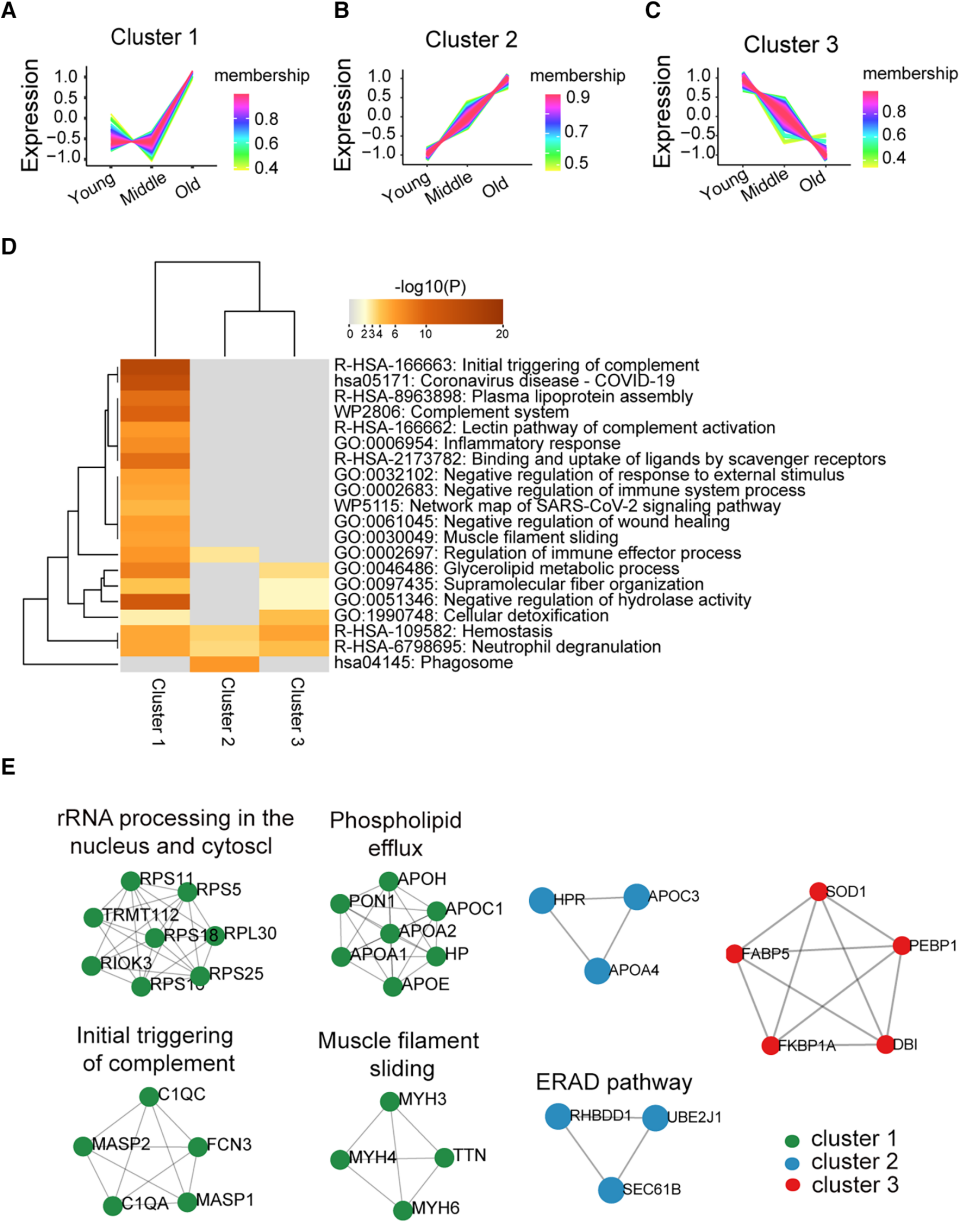
Differentially expressed proteins and biological pathways identified in the middle and old groups compared with the young group. (**A**–**C**) The protein changes were divided into three clusters according to trends from young to middle to old by C-means cluster analysis (a line represents a protein). (**D**) Pathway enrichment analysis of the three clusters of protein was performed using Metascape online analysis (the significantly enriched pathway has been defined with the input of overlap proteins ≥3, *p* < 0.01). (**E**) Detected PPI modules in clusters.

To explore the dynamic changes in the platelet proteome during ageing, we performed cluster and PPI network analyses among the young, middle, and old populations. Proteins within cluster 1 (*n* = 238) showed a steady trend in the young and middle groups but significantly increased in the old group (Figure 2A). The enriched pathways among these proteins were most associated with the initial triggering of complement (Figure 2D). Proteins within cluster 2 (*n* = 137) displayed a significant age-dependent increasing trend, progressing from the young to the middle-aged and further to the old populations. (Figure 2B); the phagosome pathway was strongly enriched in these proteins (Figure 2D). Protein expression in cluster 3 (*n* = 91) showed an age-related decreasing trend in the middle-aged and old groups (Figure 2C); these proteins were mainly involved in haemostasis, neutrophil degranulation, and cellular detoxification based on pathway enrichment analysis (Figure 2D). The PPI network based on the molecular complex detection (MCODE) algorithm analysis is shown in Figure 2E, which identified seven functional clusters. The above results suggested the potential functional changes in platelets during ageing. The list of proteins from clusters 1–3 can be found in Supplementary Data Sheet 2.

### 3.3 Comparison of the platelet proteome between the old and young groups

**Figure 3:**
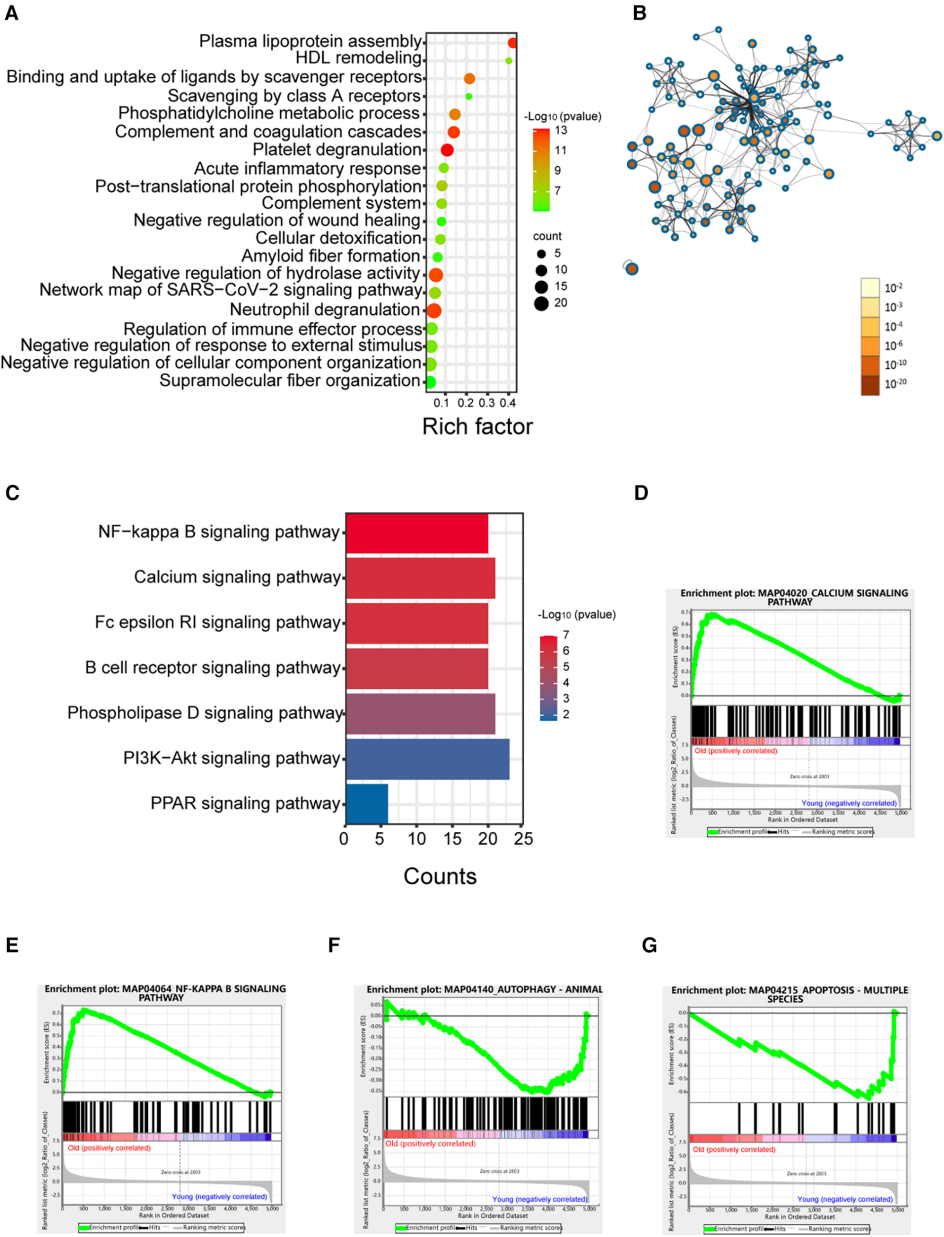
Enrichment analysis of proteins in the elderly group compared with the young group. (**A**) Enrichment dot bubble of DE proteins. (**B**) PPI network of DE proteins. The color and size of each node represent the statistical significance (*p* value). (**C**) KEGG enrichment analysis of signalling pathways of DE proteins. (**D**–**G**) GSEA snapshots of KEGG pathway enrichment analysis. DE, differential expression; PPI, protein-protein interaction; GSEA, gene set enrichment analysis.

Based on the analysis above, the largest difference in the platelet protein expression profile was seen between the elderly and young groups. To better elucidate the effect of age on the platelet proteome, we analysed the differences in the proteome between the elderly and young groups. As shown in Figure 3A, the enriched processes were “platelet degranulation”, and others. The proteins associated with platelet degranulation are shown in Table 2. PPI network analysis was performed on the differentially expressed proteins in the old group compared with the young group (Figure 3B). KEGG enrichment analysis showed seven signalling pathways in Figure 3C, most of which are associated with platelet activity and function (19, 20). Furthermore, GSEA revealed that the calcium signalling pathway and the NF-*κ*B signalling pathway were enriched in the old group (Figures 3D,E), which is consistent with the hyperactivity of platelets in elderly populations.

**Table 2:** Significantly altered proteins associated with platelet degranulation in young and old groups.

Gene name	Protein ID	Description	*p* value	Fold change	Regulation
CALU	B3KQF5	Calumenin	0.0337	3.3812	Up
TFPI	P10646	Tissue factor pathway inhibitor	0.0055	1.5325	Up
PFN1	P07737	Profilin-1	0.0014	0.6627	Down
SOD1	P00441	Superoxide dismutase [Cu-Zn]	0.0027	0.6538	Down
TMSB4X	A2VCK8	Thymosin beta	0.0008	0.6320	Down
MIF	A6MUU8	Macrophage migration inhibitory factor	0.0092	0.6580	Down
PRKAR2B	A0A024R712	Protein kinase, cAMP-dependent, regulatory, type II, beta, isoform CRA_a	0.0032	0.6082	Down

Based on the GSEA, we found that the apoptosis and autophagy pathways were negatively enriched in the old group (Figures 3F,G), which indicated that the apoptosis and autophagy functions of platelets in the elderly were decreased. In this study, we observed a significant downregulation of ATG7, as a conventional autophagy-related gene, consistent with previous research findings (Supplementary Figure S3) (21).

**Figure 4:**
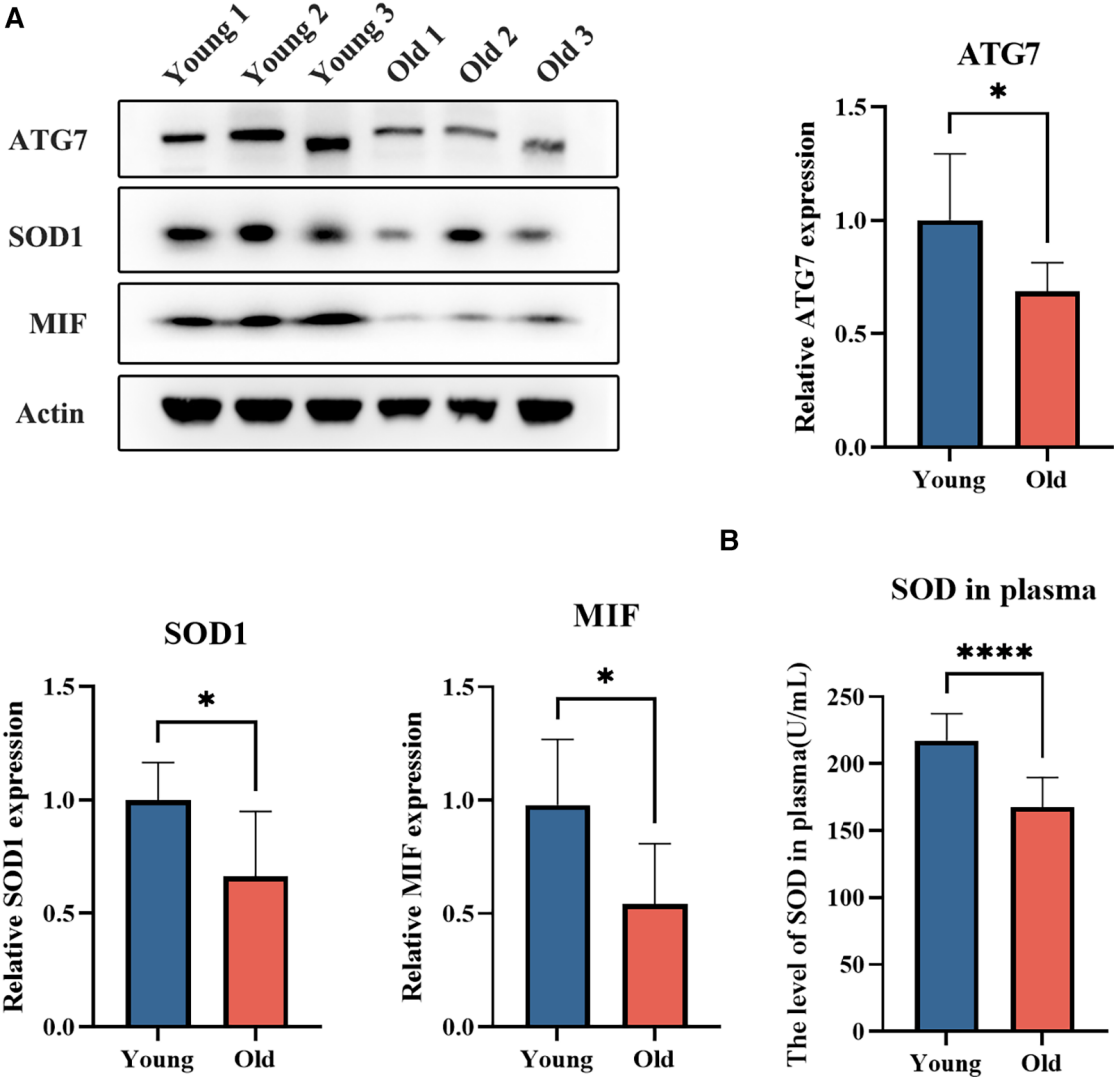
The expression levels of platelet ATG7, SOD1, and MIF, as well as the plasma SOD level in young and elderly individuals. (**A**) The expression levels of platelet ATG7, SOD1, and MIF in young and elderly individuals. (**B**) The plasma SOD levels in young and elderly individuals.

The expression levels of platelet degranulation-related proteins SOD1 and MIF, as well as autophagy-related protein ATG7, were assessed using western blot analysis in an independent cohort of young (*n* = 7, aged 30.0 ± 5.4 years) and elderly (*n* = 7, aged 77.1 ± 5.3 years) individuals (Figure 4A). The results revealed a significant decrease in the levels of ATG7, SOD1, and MIF in the elderly group (*p* < 0.05). The findings are consistent with the results obtained from DIA MS analysis. Moreover, we assessed the levels of SOD in plasma samples obtained from both young and elderly cohorts, which revealed a significant decline in SOD concentrations within the elderly group (*p* < 0.05) (Figure 4B).

### 3.4 Proteins correlated with complement activation were increased with ageing

**Figure 5:**
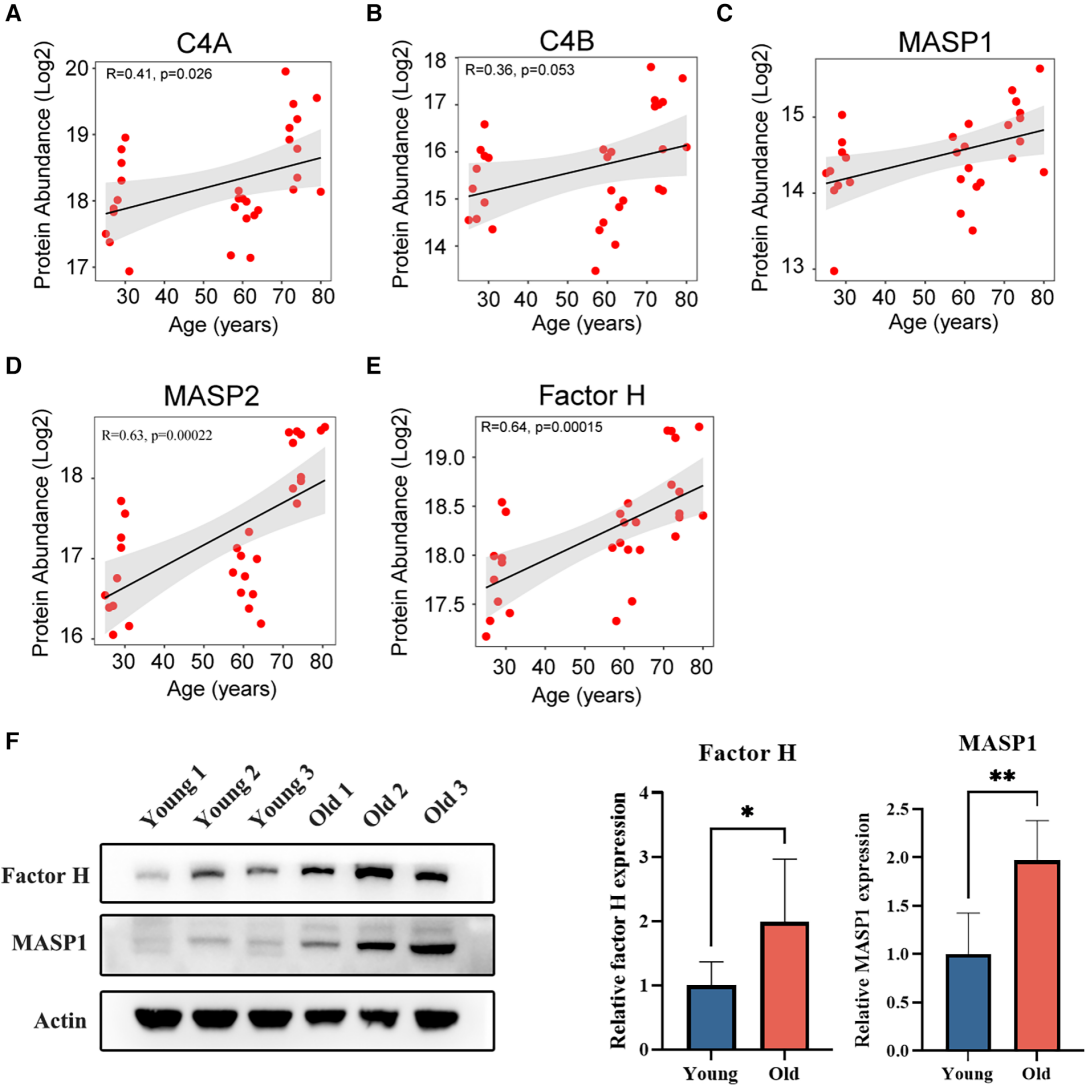
Age-related correlation analysis of proteins associated with complement activation. (**A**,**B**) Age-related correlation analysis of C4 proteins with age. (**C**,**D**) Age-related correlation analysis of MASP proteins with age. (**E**) Age-related correlation analysis of factor H with age. (**F**) The expression levels of platelet factor H and MASP1 in young and elderly individuals.

Based on the analysis above, differences in platelet proteins across various age groups may be associated with complement activation. The complement system is an important noncellular factor in the rapid response to tissue damage or inflammation that helps maintain integrity and prevent serious damage to the body. There are three pathways of complement activation, including the classical pathway, the alternative pathway and the lectin pathway. C4 is an important factor in the classical pathway, and the MASP family plays a key role in the lectin pathway (22). Factor H is the negative regulator of the alternative pathway that prevents complement consumption via uncontrolled alternative pathway activation on the surface of platelets (23). In this study, we observed a general upregulation of C4, MASP1, MASP2, and factor H expression in platelets among the elderly population (Figures 5A–E). Moreover, the expression levels of factor H and MASP1 were assessed using western blot analysis in both young and elderly groups (Figure 5F). The results revealed a significant increase in the levels of factor H and MASP1 (*p* < 0.05), which is consistent with the results obtained from DIA MS analysis.

## 4 Discussion

Ageing is a risk factor for cardiovascular disease, which is closely related to platelet hyperactivity. Thus, it is necessary to explore the mechanism of platelet hyperactivity in elderly individuals. Proteins are the main performers of cell function. The changes in the platelet proteome during ageing provide a theoretical basis for studying platelet hyperactivity and its underlying mechanisms. By using proteomics analysis, we found that the platelet proteome was relatively consistent between the young and middle-aged groups; however, when compared with the young or middle-aged groups, the elderly group exhibited more upregulation of proteins. This trend is consistent with a previous study showing that platelet counts remain stable between young and middle-aged populations (20–60 years old) but decrease in elderly populations (>70 years old) (24–26). Moreover, the enrichment analysis of common differentially expressed proteins in the old vs. young and middle vs. young groups showed that these common differentially expressed proteins were mainly related to platelet production. This suggests that the proteome of platelets can reflect the effect of ageing on platelets.

The endoplasmic reticulum-associated degradation (ERAD) pathway is an important protein quality control system that helps maintain protein homeostasis (27). Disruption of protein homeostasis has been identified as a marker of ageing, but no studies have yet shown the role of ERAD in ageing. In this study, we found that proteins, including RHBDD1, UBE2J1, and SEC61B, which increase with age, are associated with the ERAD pathway, as determined by the MCODE algorithm. This may provide insight into the effects of ageing on platelet protein homeostasis. However, additional research is required to investigate the impact of ERAD-related proteins on platelet function and its correlation with platelet hyperactivity.

Additionally, platelet degranulation was the most relevant process in the differentially expressed protein enrichment analysis between the young and the aged groups. This finding may explain the reason for platelet hyperactivity in elderly individuals through changes in protein profiles. The development of therapies targeting platelet degranulation-associated proteins to attenuate platelet hyperactivity in the elderly may contribute to a reduction in the risk of hyperthrombotic disease. In recent years, autophagy proteins have been detected in platelets and demonstrated to be involved in platelet aggregation, adhesion, and thrombus formation (28). Autophagy is an essential cellular process that mediates the degradation of proteins and organelles in lysosomes and has been tightly linked to cellular quality control due to its role as part of the proteostasis network (29). Autophagy can clear the inflammasome and its upstream-triggered proteins; thus, the reduction in autophagic flux may enhance inflammation (30). A study reported that the expression of autophagy-related genes, such as autophagy-related protein 5 (ATG5), ATG7, and beclin 1 (BECN1), declines with age in humans (21). A recent article also reported the dysregulation of autophagy in aged platelets (31). This is consistent with our research. Platelet apoptosis and autophagy may be decreased in elderly individuals according to the GSEA performed in the present study. Furthermore, studies have suggested a strong association between apoptosis and ageing (29, 32), however, the interplay between ageing and apoptosis has proven complex. Therefore, the effects of apoptosis on platelet function during ageing need further study.

Complement activation was most enriched in the shared differentially expressed proteins of the old vs. young and the old vs. middle groups in the enrichment analysis. The complement system is comprised of a series of proteases and inhibitors that are activated in a cascade-like fashion during host defence (33). At the site of vascular damage, platelets and platelet-derived microparticles can promote complement activation, which is involved in vascular inflammation. In this study, the levels of C4, MASP1, and MASP2 and the level of factor H were higher in platelets in the elderly group than in the young and middle-aged groups. Complement C4 precursor proteins are secreted from platelet alpha-granules, which can participate in the activation of the complement classical pathway and further promote the activation of platelets (34). The activation of C4 not only led to the activation of the complement classical pathway but also enhanced platelet activation. A recent study reported that the MASP family is activated during coagulation (35), which is associated with platelet activation and may be a novel mechanism of complement-platelet crosstalk that triggers thrombo-inflammation (35). Factor H is particularly abundant on platelets (36). Previous research has reported that factor H may have an antithrombotic role because it may inhibit blood coagulation through its platelet receptor (23). Therefore, the increase in factor H may be a compensatory mechanism in elderly individuals, which may prevent excessive platelet activation and thus decrease the risk of thrombotic diseases. In general, these complement proteins can also interact with platelets, thereby affecting platelet activation, which suggests that the elevation of these complement proteins in platelets might be one of the reasons for platelet hyperreactivity in elderly individuals.

There are some limitations in the study. Firstly, the sample size of this study is limited to only 30 samples. Since this is a small exploratory study, it may also provide insights into the impact of ageing on the platelet proteome. Secondly, we used a classical approach based on differential centrifugation for platelet isolation, inevitably resulting in a population of platelets contaminated by other circulating cells. Thirdly, we did not conduct functional evaluation of the platelets used in this study. These aspects require further improvement in our future research.

## 5 Conclusion

In this study, we revealed that the platelet proteome changed with ageing, especially in elderly individuals. Enrichment analysis showed that the differentially expressed proteins were associated with platelet degranulation and complement activation. Additionally, the decline in autophagy in the elderly population indicates the disruption of platelet proteostasis. This novel evidence has important implications for revealing changes in platelet function that contribute to platelet hyperactivity and the susceptibility to thrombosis in elderly individuals.

## Data Availability

The mass spectrometry proteomics data have been deposited to the ProteomeXchange Consortium (https://proteomecentral.proteomexchange.org) via the iProX partner repository (37, 38) with the dataset identifier PXD048996.

## References

[1] van der Meijden PEJ Heemskerk JWM. Platelet biology and functions: new concepts and clinical perspectives. Nat Rev Cardiol. (2019) 16(3):166–79. 10.1038/s41569-018-0110-0 30429532

[2] Garraud O Cognasse F. Are platelets cells? And if yes, are they immune cells? Front Immunol. (2015) 6:70. 10.3389/fimmu.2015.00070 25750642 PMC4335469

[3] Donato AJ Machin DR Lesniewski LA. Mechanisms of dysfunction in the aging vasculature and role in age-related disease. Circ Res. (2018) 123(7):825–48. 10.1161/circresaha.118.312563 30355078 PMC6207260

[4] da Costa Martins P García-Vallejo JJ van Thienen JV Fernandez-Borja M van Gils JM Beckers C P-selectin glycoprotein ligand-1 is expressed on endothelial cells and mediates monocyte adhesion to activated endothelium. Arterioscler Thromb Vasc Biol. (2007) 27(5):1023–9. 10.1161/atvbaha.107.140442 17322099

[5] Langer HF Gawaz M. Platelet-vessel wall interactions in atherosclerotic disease. Thromb Haemost. (2008) 99(3):480–6. 10.1160/th07-11-0685 18327395

[6] Montenont E Rondina MT Campbell RA. Altered functions of platelets during aging. Curr Opin Hematol. (2019) 26(5):336–42. 10.1097/moh.0000000000000526 31348047 PMC7039178

[7] Kasjanovová D Baláz V. Age-related changes in human platelet function in vitro. Mech Ageing Dev. (1986) 37(2):175–82. 10.1016/0047-6374(86)90074-6 3821196

[8] Gnanenthiran SR Pennings GJ Reddel CJ Campbell H Kockx M Hamilton JR Identification of a distinct platelet phenotype in the elderly: ADP hypersensitivity coexists with platelet PAR (protease-activated receptor)-1 and PAR-4-mediated thrombin resistance. Arterioscler Thromb Vasc Biol. (2022) 42(8):960–72. 10.1161/atvbaha.120.316772 35708029

[9] Bastyr EJ 3rd Kadrofske MM Vinik AI. Platelet activity and phosphoinositide turnover increase with advancing age. Am J Med. (1990) 88(6):601–6. 10.1016/0002-9343(90)90525-i 2161185

[10] Le Blanc J Lordkipanidzé M. Platelet function in aging. Front Cardiovasc Med. (2019) 6:109. 10.3389/fcvm.2019.00109 31448291 PMC6692461

[11] Winkler W Zellner M Diestinger M Babeluk R Marchetti M Goll A Biological variation of the platelet proteome in the elderly population and its implication for biomarker research. Mol Cell Proteomics. (2008) 7(1):193–203. 10.1074/mcp.M700137-MCP200 17962630

[12] Pagel O Walter E Jurk K Zahedi RP. Taking the stock of granule cargo: platelet releasate proteomics. Platelets. (2017) 28(2):119–28. 10.1080/09537104.2016.1254762 27928935

[13] Zeiler M Moser M Mann M. Copy number analysis of the murine platelet proteome spanning the complete abundance range. Mol Cell Proteomics. (2014) 13(12):3435–45. 10.1074/mcp.M114.038513 25205226 PMC4256495

[14] Burkhart JM Vaudel M Gambaryan S Radau S Walter U Martens L The first comprehensive and quantitative analysis of human platelet protein composition allows the comparative analysis of structural and functional pathways. Blood. (2012) 120(15):e73–82. 10.1182/blood-2012-04-416594 22869793

[15] Yu H Liu Y He B He T Chen C He J Platelet biomarkers for a descending cognitive function: a proteomic approach. Aging Cell. (2021) 20(5):e13358. 10.1111/acel.13358 33942972 PMC8135080

[16] Sabrkhany S Kuijpers MJE Knol JC Olde Damink SWM Dingemans AC Verheul HM Exploration of the platelet proteome in patients with early-stage cancer. J Proteomics. (2018) 177:65–74. 10.1016/j.jprot.2018.02.011 29432918

[17] Bereczki E Branca RM Francis PT Pereira JB Baek JH Hortobágyi T Synaptic markers of cognitive decline in neurodegenerative diseases: a proteomic approach. Brain. (2018) 141(2):582–95. 10.1093/brain/awx352 29324989 PMC5837272

[18] Yu H Liu Y He T Zhang Y He J Li M Platelet biomarkers identifying mild cognitive impairment in type 2 diabetes patients. Aging Cell. (2021) 20(10):e13469. 10.1111/acel.13469 34528736 PMC8520722

[19] Ye RD Kravchenko VV Pan Z Feng L. Stimulation of NF-kappa B activation and gene expression by platelet-activating factor. Adv Exp Med Biol. (1996) 416:143–51. 10.1007/978-1-4899-0179-8_24 9131140

[20] Wan P Tan X Xiang Y Tong H Yu M. PI3K/AKT and CD40l signaling regulate platelet activation and endothelial cell damage in sepsis. Inflammation. (2018) 41(5):1815–24. 10.1007/s10753-018-0824-5 29956071

[21] Lipinski MM Zheng B Lu T Yan Z Py BF Ng A Genome-wide analysis reveals mechanisms modulating autophagy in normal brain aging and in Alzheimer’s disease. Proc Natl Acad Sci U S A. (2010) 107(32):14164–9. 10.1073/pnas.1009485107 20660724 PMC2922576

[22] Nording H Langer HF. Complement links platelets to innate immunity. Semin Immunol. (2018) 37:43–52. 10.1016/j.smim.2018.01.003 29426568

[23] Ferluga J Kishore U Sim RB. A potential anti-coagulant role of complement factor H. Mol Immunol. (2014) 59(2):188–93. 10.1016/j.molimm.2014.02.012 24632373

[24] Vázquez-Santiago M Ziyatdinov A Pujol-Moix N Brunel H Morera A Soria JM Age and gender effects on 15 platelet phenotypes in a Spanish population. Comput Biol Med. (2016) 69:226–33. 10.1016/j.compbiomed.2015.12.023 26773944

[25] Troussard X Vol S Cornet E Bardet V Couaillac JP Fossat C Full blood count normal reference values for adults in France. J Clin Pathol. (2014) 67(4):341–4. 10.1136/jclinpath-2013-201687 24170208

[26] Segal JB Moliterno AR. Platelet counts differ by sex, ethnicity, and age in the United States. Ann Epidemiol. (2006) 16(2):123–30. 10.1016/j.annepidem.2005.06.052 16246584

[27] Zhu B Jiang L Huang T Zhao Y Liu T Zhong Y ER-associated degradation regulates Alzheimer’s amyloid pathology and memory function by modulating *γ*-secretase activity. Nat Commun. (2017) 8(1):1472. 10.1038/s41467-017-01799-4 29133892 PMC5684335

[28] Schwertz H Middleton EA. Autophagy and its consequences for platelet biology. Thromb Res. (2023) 231:170–81. 10.1016/j.thromres.2022.08.019 36058760 PMC10286736

[29] Kaushik S Tasset I Arias E Pampliega O Wong E Martinez-Vicente M Autophagy and the hallmarks of aging. Ageing Res Rev. (2021) 72:101468. 10.1016/j.arr.2021.101468 34563704 PMC8616816

[30] Deretic V Kroemer G. Autophagy in metabolism and quality control: opposing, complementary or interlinked functions? Autophagy. (2022) 18(2):283–92. 10.1080/15548627.2021.1933742 34036900 PMC8942406

[31] de Sousa DMB Poupardin R Villeda SA Schroer AB Fröhlich T Frey V The platelet transcriptome and proteome in Alzheimer’s disease and aging: an exploratory cross-sectional study. Front Mol Biosci. (2023) 10:1196083. 10.3389/fmolb.2023.1196083 37457829 PMC10348715

[32] Zhang JH Zhang Y Herman B. Caspases, apoptosis and aging. Ageing Res Rev. (2003) 2(4):357–66. 10.1016/s1568-1637(03)00026-6 14522240

[33] Makrides SC. Therapeutic inhibition of the complement system. Pharmacol Rev. (1998) 50(1):59–87. 9549758

[34] Maynard DM Heijnen HF Horne MK White JG Gahl WA. Proteomic analysis of platelet alpha-granules using mass spectrometry. J Thromb Haemost. (2007) 5(9):1945–55. 10.1111/j.1538-7836.2007.02690.x 17723134

[35] Kozarcanin H Lood C Munthe-Fog L Sandholm K Hamad OA Bengtsson AA The lectin complement pathway serine proteases (MASPs) represent a possible crossroad between the coagulation and complement systems in thromboinflammation. J Thromb Haemost. (2016) 14(3):531–45. 10.1111/jth.13208 26614707

[36] Vaziri-Sani F Hellwage J Zipfel PF Sjöholm AG Iancu R Karpman D. Factor H binds to washed human platelets. J Thromb Haemost. (2005) 3(1):154–62. 10.1111/j.1538-7836.2004.01010.x 15634279

[37] Ma J Chen T Wu S Yang C Bai M Shu K Iprox: an integrated proteome resource. Nucleic Acids Res. (2019) 47(D1):D1211–7. 10.1093/nar/gky869 30252093 PMC6323926

[38] Chen T Ma J Liu Y Chen Z Xiao N Lu Y Iprox in 2021: connecting proteomics data sharing with big data. Nucleic Acids Res. (2022) 50(D1):D1522–d1527. 10.1093/nar/gkab1081 34871441 PMC8728291

