# Supplementary material for: Identification of the changes in the platelet proteomic profile of elderly individuals
Source: Front Cardiovasc Med. 2024 May 14;11:1384679. doi: 10.3389/fcvm.2024.1384679 (PMC11130443; doi:10.3389/fcvm.2024.1384679)
Supplement: Supplementary file 3 [file Datasheet3.docx]

Supplementary Material

# Supplementary Figures


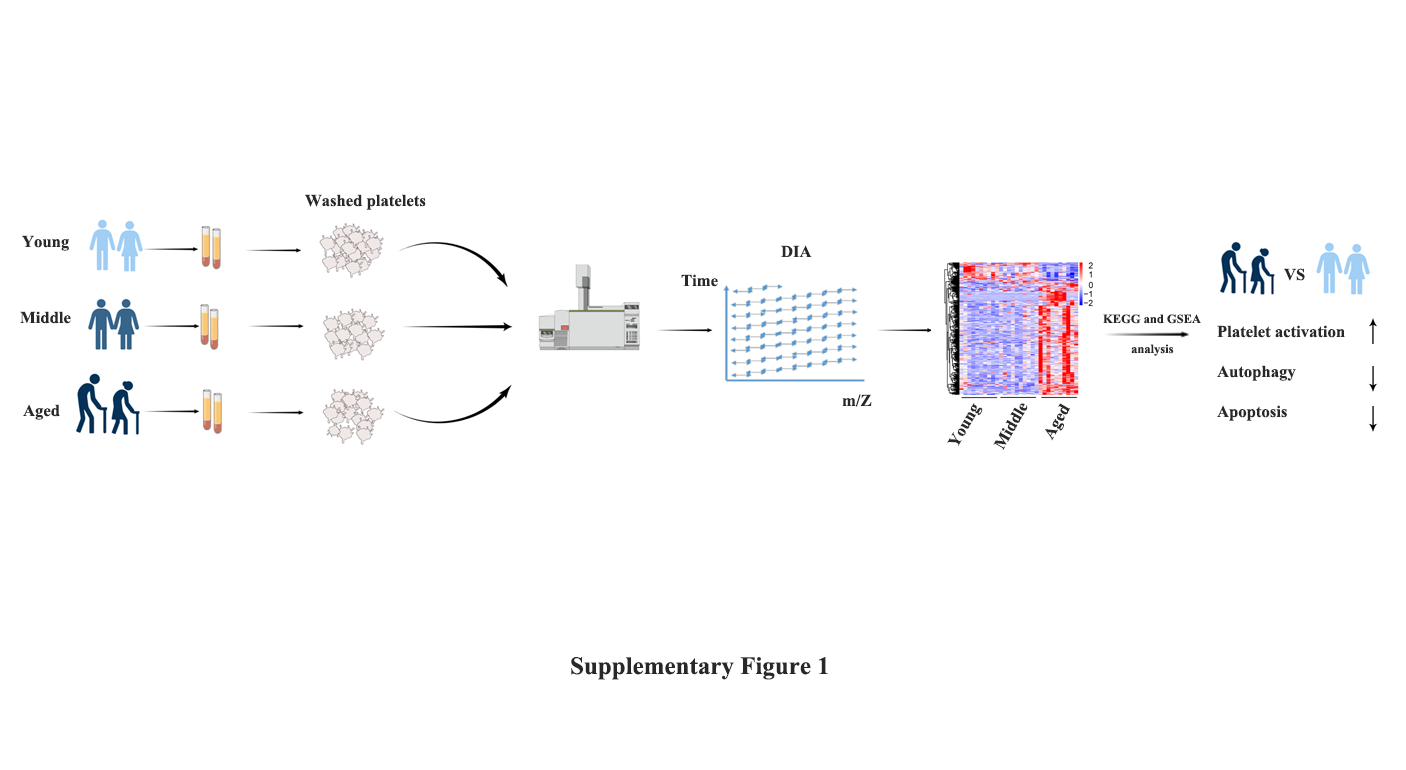


**Supplementary Figure 1.** Flow chart


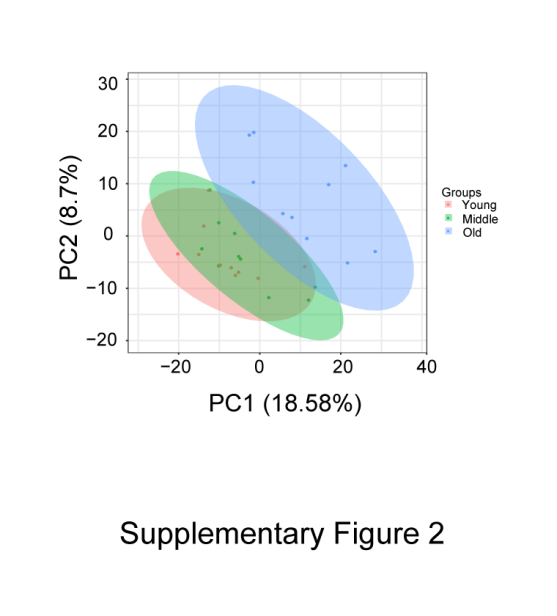


**Supplementary Figure 2.** Principal component analysis (PCA) based on the proteomic data showing the separation of groups by age.


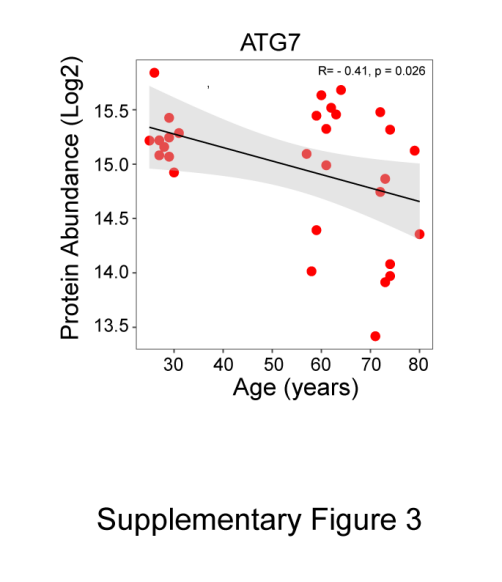


**Supplementary Figure 3.** Correlation analysis of ATG7 with age.
